# Does integrated community case management (iCCM) target health inequities and treatment delays? Evidence from an analysis of Demographic and Health Surveys data from 21 countries in the period 2010 to 2018

**DOI:** 10.7189/jogh.11.04013

**Published:** 2021-03-01

**Authors:** Peter Winskill, Andria Mousa, Olusola Oresanya, Helen Counihan, Lucy C Okell, Patrick G Walker

**Affiliations:** 1 MRC Centre for Global Infectious Disease Analysis, Imperial College, London; 2 Malaria Consortium, Abuja, Nigeria; 3 Malaria Consortium, London, UK

## Abstract

**Background:**

Integrated community case management (iCCM) is a programme that can, via community health workers (CHWs), increase access to timely and essential treatments for children. As well as improving treatment coverage, iCCM has an additional equity-focus with the aim of targeting underserved populations. To assess the success of iCCM programmes it is important that we understand the contribution they are making to equitable health coverage.

**Methods:**

We analysed demographic and health survey data from 21 countries over 9 years to assess evidence and evaluate iCCM programmes. We summarise the contribution CHWs are making relative to other health care provider groups and what treatment combinations CHWs are commonly prescribing. We assessed the ability of CHWs to target treatment delays and health inequities by evaluating time to treatment following fever onset and relationships between CHWs and wealth, rurality and remoteness.

**Results:**

There was good evidence that CHWs are being successfully targeted to improve inequities in health care coverage. There is a larger contribution of CHWs in areas with higher poverty, rurality and remoteness. In six surveys CHWs were associated with significantly shorter average time between fever onset and advice or treatment seeking, whilst in one they were associated with significantly longer times. In areas with active CHW programmes, the contribution of CHWs relative to other health care provider groups varied between 11% to 45% of treatment visits. The distribution of types of treatment provided by CHWs was also very variable between countries.

**Conclusions:**

The success of an iCCM programme depends not only on increasing treatment coverage but addressing inequities in access to timely health care. Whilst much work is still needed to attain universal health care targets, and despite incomplete data, there is evidence that iCCM is successfully addressing treatment delays and targeting underserved populations.

Integrated community case management (iCCM) is a strategy to improve access to essential treatment services for children through the use of community health workers (CHWs) (1). Under the iCCM approach CHWs are provided with the training, supplies and supervision to diagnose, treat or refer children presenting with the major causes of deaths in under-fives: pneumonia, diarrhoea, malaria and newborn conditions. In addition, most CHW programmes also screen for acute malnutrition, as indicated by the mid-upper arm circumference (MUAC). As well as aims to improve treatment coverage and delays to treatment, at the heart of the iCCM strategy is a focus on equity [1]. This pro-equity approach aims to extend health care access to those living in underserved areas at the limits or outside of the formal health care system [2].

There are a multitude of challenges associated with extending health care to underserved populations, which, by their nature, are often the hardest to reach. There is a strong need for evidence to inform future efforts and to assess and regulate the quality of iCCM [3], goals which are hampered by often weak or under-resourced monitoring and evaluation systems in the counties or regions where iCCM is implemented [4]. Many low to middle income countries (LICs and LMICs) worldwide, from Afghanistan to Zimbabwe [5], have adopted iCCM, and whilst core elements are similar, the extent, scope and implementation of programmes vary considerably. This policy and programmatic fragmentation make policy-level assessments of iCCM difficult. Furthermore, in many instances, especially in sun-Saharan Africa (SSA), CHW programmes are sub-nationally implemented and are associated with partner support and as such, coverage and sustainability goals have not been reliably met [6]. Large scale multi-country implementations, such as the rapid access expansion programme (RAcE) have been undertaken, although these attempted to align with government policies so context specific differences were still present. The RAcE programme aimed to assess impact and catalysed scale-up and policy updates in five countries in sub-Saharan Africa [7], and demonstrated a strong impact on levels of care-seeking from an appropriate provider for sick children (8). In order to assess the success of iCCM service delivery aims it is important to understand the impact that iCCM has had not only on treatment coverage, but on equity-focussed and time-to-treatment aspects of the strategy as well.

Whilst clinical trials and within country monitoring and evaluation of programmes may be the gold standard for assessing iCCM aims, there are other large data resources that can be additionally leveraged to inform our understanding of iCCM across multiple countries and years. One such resource is the demographic and health surveys (DHS) program [8]. This is a standardised, nationally representative set of household surveys conducted at regular intervals across many countries. DHS data includes measures relating to child health, treatment seeking and treatment coverage which can all be linked at the individual level. DHS data can also provide information on or be linked to known measures of health inequity – delays to treatment/care seeking [9,10], wealth [11], rurality [12] and remoteness [13] – which are all associated with poor access to care and negative health outcomes. As such, whilst not focussed on iCCM, the DHS surveys can provide us with an important window onto many of the aspects of health care access that iCCM addresses including, importantly, populations not covered by iCCM.

In this study we have analysed DHS surveys from 21 low- and middle-income countries over 10 years. We present information on the contribution CHWs are making to health care provision within these countries, comparing CHWs with other health care providers and assessing the treatments CHWs are providing. For a subset of countries and years we analyse the success of the equity-focussed component of iCCM, assessing trends in treatment delays and CHW contributions with respect to wealth, rurality and remoteness.

## METHODS

We collated data from all DHS surveys in LMICs from 2010 to 2019 (full data sets are available to registered users) [8] using the rDHS package [14]. Of these, a subset was selected on the basis of containing identifiable CHW information in sections relating to health care providers and were used for the analysis. From these data sets we extracted information relating to childhood illnesses, treatment seeking, treatment providers, treatment types and equity:

1. Childhood illnesses: has the child had fever/cough or diarrhoea in the two weeks preceding the survey?
2. Treatment seeking: was treatment sought? How many days after onset of symptoms was advice or treatment for fever obtained?
3. Treatment provider: recorded provider for each treatment sought.
4. Diagnosis and treatments: which treatments were given - artemisinin-based combination therapies (ACTs), antibiotic, oral rehydration salts (ORS), recommended homemade fluids (RHF) or zinc? Was blood taken from a finger or heel for malaria testing (referred to as “Test”)?
5. Equity: household wealth index, survey cluster location: rural/urban, survey cluster GIS coordinates.

One further equity measure, remoteness, was also estimated using the cluster-level coordinates and a published surface of travel times to the nearest city or large urban centre [13]. For each cluster, the mean travel time within a 5km buffer of the published coordinates was estimated, to account for the anonymisation procedure of GIS data.

Treatment provider classifications were aggregated into five broad groups to standardise the 256 unique treatment provider types that were identified across all surveys and included locally relevant terms where possible and are detailed in the provider classification in the **Online Supplementary Document**. The five provider groups were CHW, public, private, traditional and other. Where more than one treatment provider was listed (7%), the one reported first was used.

We estimated the proportion of treatment seeking visits attributed to each of the five provider groups for each country/y survey in clusters (DHS sample unit) where CHW activity could be identified as well as at the national level. We compared the frequency that CHW, public and private sector visits resulted in each diagnostic or treatment combination being used or prescribed. To assess the relationship between CHW use and wealth we estimated the probability that a treatment visit would be from a CHW with respect to wealth index, rurality and remoteness.

Mean estimates were calculated using the cluster-level survey weighting and presented with bootstrapped 95% confidence intervals (CI). To estimate intervals of the mean estimates, we take the 2.5th and 97.5th percentiles of the distribution of weighted means from 1000 resampled data sets. As a result, estimates with zero variance were not assigned CIs. Hypothesis testing (difference between mean estimates) was also performed using bootstrap *t* tests to account for small sample sizes and non-normal distributions in a number of instances [15]. All data processing, analysis and visualisation was performed in R [16].

## RESULTS

We collated survey data from 21 countries over 9 years that contained identifiable information regarding CHWs. These data contained records for 560 546 children, of whom 121 482 were recorded as having sought treatment for an illness in the preceding two weeks before a survey and 2538 were recorded as having sought treatment from a CHW.

### What is the relative contribution of CHWs compared to other treatment providers?

As a percentage of all treatment seeking records, treatment seeking from a CHW (in clusters where CHWs activity was identified) constituted on average around one quarter (24.7%, 95% CI = 23.8%, 25.6%) of all treatment seeking visits (**Table 1**, Table S1 in the **Online Supplementary Document**), although the between country and year variation was large, ranging from 11.1% of recorded treatment seeking visits in Pakistan in 2017 to 44.8% in Madagascar in 2016. Assessed at the country-level, the percentage of treatment seeking visits to a CHW was much lower (2.1%, 95% CI = 2.0%, 2.2%) (Table S2 in the **Online Supplementary Document**), a reflection of the highly targeted nature of most CHW programmes.

**Table 1:** The percentage of treatment visits assigned to each health care provider group for the most recent demographic and health survey in each country*

Country	Year	N	Percentage of visits from each provider (95% CI)				
**CHW**	**Public**	**Private**	**Traditional**	**Other**
**Benin**	2012	49	31.2 (17.6, 45.3)	41.4 (27.5, 56.8)	18.6 (8.7, 29.3)	8.8 (2, 16.9)	0 (-, -)
**Burkina Faso**	2014	183	15.3 (9.9, 20.7)	71.8 (65.1, 78.4)	7.6 (3.8, 11.8)	4.7 (1.8, 8.1)	0.6 (0, 1.8)
**Burundi**	2016	709	18.7 (15.9, 21.6)	74.3 (70.9, 77.8)	6 (4.3, 7.9)	0.6 (0, 1.4)	0.4 (0, 1)
**DRC**	2013	178	10.3 (4.8, 16.9)	53.1 (43.3, 63.3)	27.9 (18.9, 37.5)	7.5 (2.7, 12.7)	1.2 (0, 3.9)
**India**	2015	761	30.6 (26.4, 35)	26.8 (22.8, 31)	39.7 (34.9, 44.1)	0.8 (0, 1.8)	2.2 (1.1, 3.6)
**Kenya**	2014	67	28.5 (14.6, 43.6)	27.7 (14.8, 42.4)	36.6 (22.8, 51.7)	0 (-, -)	7.2 (0, 15.5)
**Lesotho**	2014	70	36.2 (23.7, 51.1)	44.5 (29.1, 58.4)	17.7 (8.6, 28.1)	0 (-, -)	1.7 (0, 4.3)
**Madagascar**	2016	163	44.8 (35.5, 54)	42.5 (33.3, 52)	7.9 (3, 13.8)	1.6 (0.1, 3.7)	3.2 (0, 8.5)
**Malawi**	2015	1325	20.3 (17.9, 22.8)	63.1 (60.2, 66)	12.8 (10.7, 14.9)	0 (0, 0.1)	3.8 (2.6, 5)
**Mali**	2018	257	24.3 (18.9, 30.4)	19.5 (14.4, 25)	46.1 (39, 53.2)	7.3 (3.7, 11.3)	2.8 (0.8, 5.3)
**Mozambique**	2011	121	37.9 (26.7, 49.1)	54.2 (42.6, 66)	1.1 (0, 3.6)	0.7 (0, 2.4)	6.1 (1.5, 12.5)
**Myanmar**	2016	141	36.4 (26, 47.3)	26.4 (18.1, 35.7)	27.5 (17.2, 38.3)	1.4 (0, 3.8)	8.2 (2.5, 15.1)
**Nigeria**	2018	713	12.5 (9.4, 15.6)	36.1 (32.2, 40.1)	49.8 (45.7, 54)	1 (0.2, 2)	0.6 (0.2, 1)
**Pakistan**	2017	168	11.1 (5.7, 18.1)	15.4 (9.3, 22.7)	67.5 (58.3, 76.4)	0 (-, -)	5.9 (1.7, 11)
**Rwanda**	2015	777	40.3 (36.5, 44)	45.2 (41.6, 49)	10.2 (7.8, 12.5)	1 (0.3, 1.7)	3.4 (2.2, 4.7)
**Senegal**	2012	109	13.9 (6.8, 22.9)	65.9 (54.5, 77)	15.8 (6.9, 25.7)	4 (0, 9.2)	0.5 (0, 1.6)
**Sierra Leone**	2013	385	17.1 (12.5, 21.6)	73 (67.8, 78.1)	5.2 (3, 7.9)	2.5 (1, 4.1)	2.2 (0.7, 3.9)
**Timor-Leste**	2016	48	43 (24.9, 61.7)	51.6 (34, 68.8)	3.6 (0, 11.5)	1.8 (0, 6.4)	0 (-, -)
**Togo**	2017	175	27.2 (20.4, 34.4)	12.8 (7.3, 18.7)	53.1 (45.6, 60.3)	3.8 (1.4, 6.5)	3.1 (0.8, 5.9)
**Uganda**	2016	1189	20 (17.3, 22.9)	40.1 (36.7, 43.7)	39.1 (35.6, 42.8)	0.5 (0.1, 1.1)	0.3 (0, 0.6)
**Zambia**	2013	379	28.2 (23.5, 33)	60.6 (55.5, 65.8)	7.1 (4.1, 10.4)	2.5 (0.8, 4.5)	1.6 (0.3, 3.3)

CHW – community health worker, CI – confidence interval

*Only clusters where community health worker activity was identified were included. Values are survey-weighted percentages with 95% weighted-bootstrapped confidence intervals.

### What treatments are CHW providing and in what combination?

**Figure 1:**
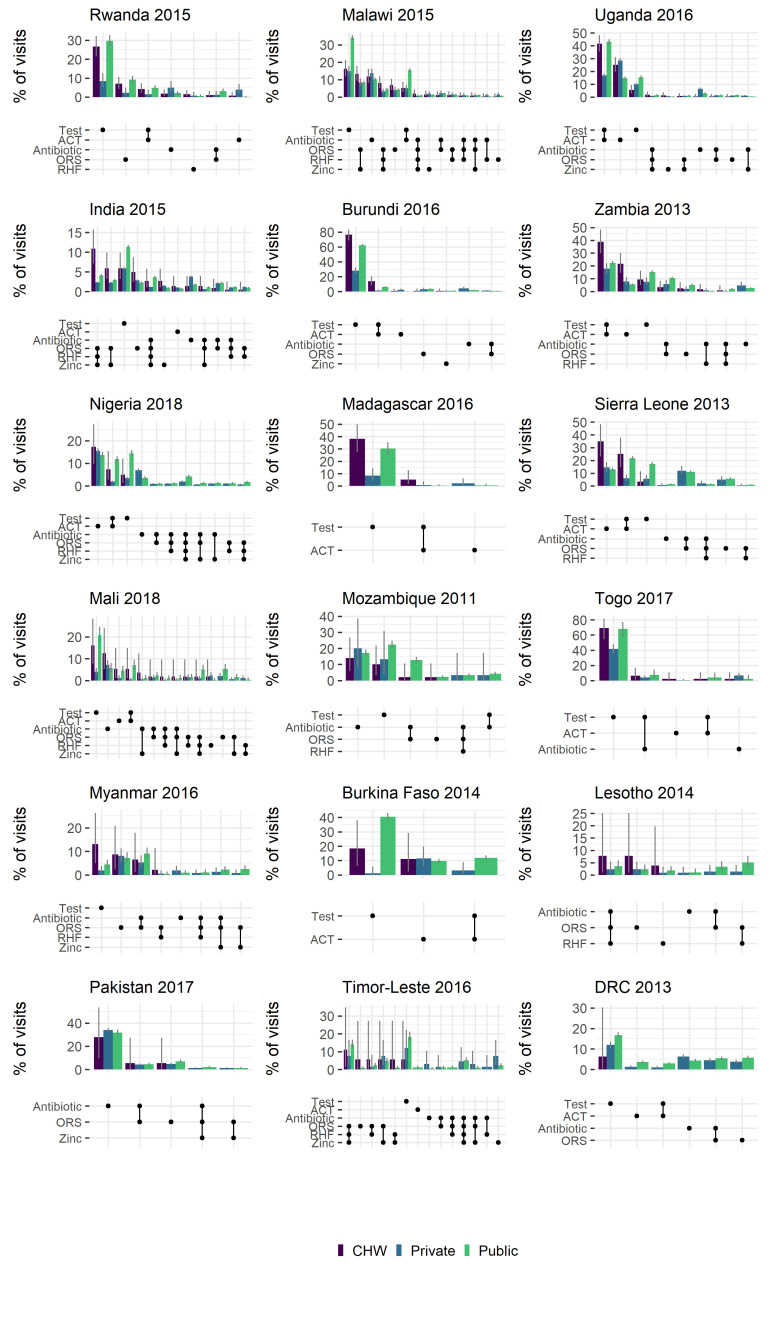
Percentage of visits resulting in single or combinations of treatments by different health care providers. Bars indicate the proportion of all visits (to a given provider) that resulted in the provision of the diagnostic(s) and/or treatment(s) shown in the lower dot-plot. Bar colours indicate provider, grey lines indicate 95% bootstrapped confidence intervals. Combinations with <1% of share are not plotted. Groupings of treatments are ordered by the % of visits to a community health worker resulting in the combination.

We estimated the proportion of treatment visits to a CHW, public or private provider that resulted in particular treatments, or combinations of treatments being provided (**Figure 1**). This provides some indication of the differing scope of CHW activity across countries, with a broad split between apparent focus on diarrhoeal disease (eg, India, Timor Leste) and those focussing on malaria (eg, Uganda, Burundi). Other general trends show some adherence to the recommendation to malaria test prior to providing an ACT (ACT is more often paired with a test, than without), at levels comparable to the public sector and consistently higher than the private sector.

### Are CHWs successful in being equity-focussed?

**Figure 2:**
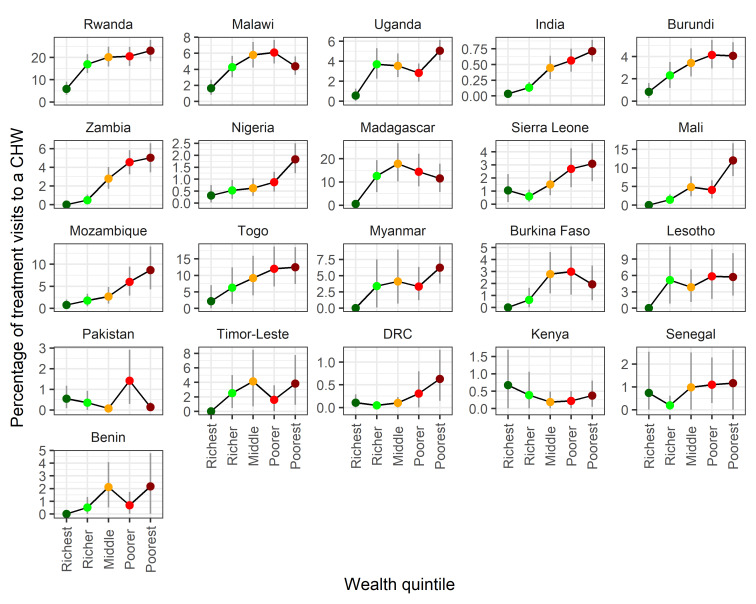
The percentage of treatment visits with a community health worker (CHW) as a function of Demographic and Health Survey wealth quintile. There is an increasing trend in the percentage of treatment visits with a CHW as poverty increases. Kenya appears to be an exception, although the results are underpowered to be certain of the visual trend. Values are survey-weighted mean estimates with 95% weighted-bootstrapped confidence intervals.

We estimated the relative contribution of visits to CHWs with respect to several equity measures. Trends with respect to wealth quintile, in general, showed an increased likelihood in treatment visits being to a CHW in households with a lower wealth index (**Figure 2**), indicating that CHWs are successful in targeting the poorest households. Trends remained largely consistent across multiple survey years (Figure S1 in the **Online Supplementary Document**).

**Figure 3:**
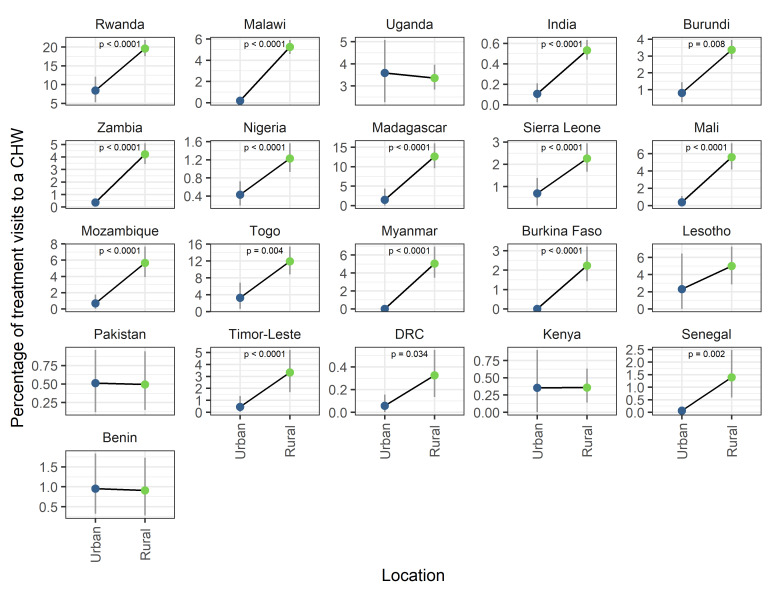
The percentage of treatment visits to a community health worker (CHW) as a function of urban or rural location. Visits to CHWs constitute a higher percentage of treatment visits in rural locations compared to urban locations. Values are survey-weighted mean estimates with 95% weighted-bootstrapped confidence intervals. *P* values indicate significantly different mean proportions between groups as determined by bootstrapped hypothesis test.

There was a significantly higher proportion of visits to CHWs in rural compared to urban areas in 16 of 21 surveys considered (**Figure 3**). For no country was there a significantly higher proportion of visits to CHWs in urban areas. Year on year trends were largely stable except for changes observed in Nigeria and Senegal, where rural targeting increased over time (Figure S2 in the **Online Supplementary Document**).

**Figure 4:**
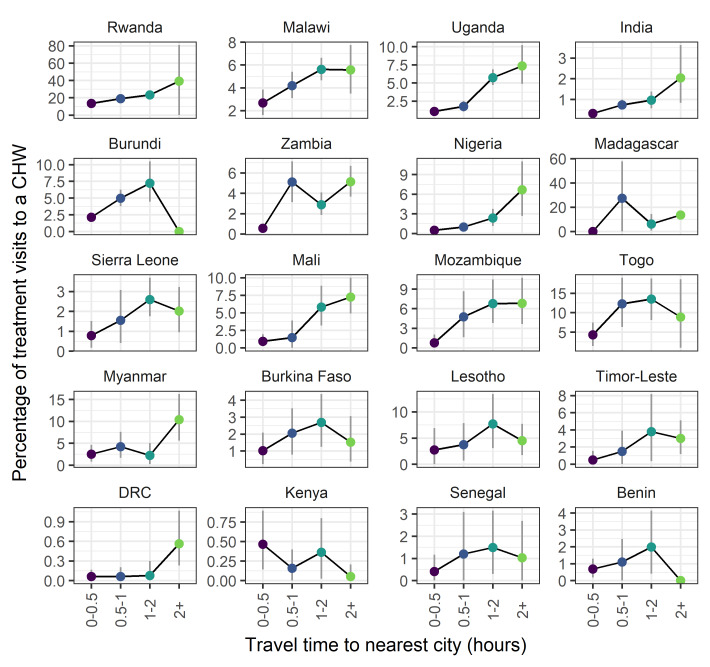
The percentage of treatment visits to a community health worker (CHW) as a function of remoteness (travel time to the nearest city). Values are survey-weighted mean estimates with 95% weighted-bootstrapped confidence intervals. For a number of countries there is an increasing trend in the percentage of treatment visits with a CHW as remoteness increases.

Trends with respect to travel time to the nearest city or large urban area were less consistent, however a number of countries did show increasing trends in the proportion of visits to CHWs with respect to travel time (eg, Rwanda, Malawi, Uganda, India) (**Figure 4**). Year on year trends within country were qualitatively similar (Figure S3 in the **Online Supplementary Document**).

### Are CHWs associated with reduced treatment delays for fevers?

**Figure 5:**
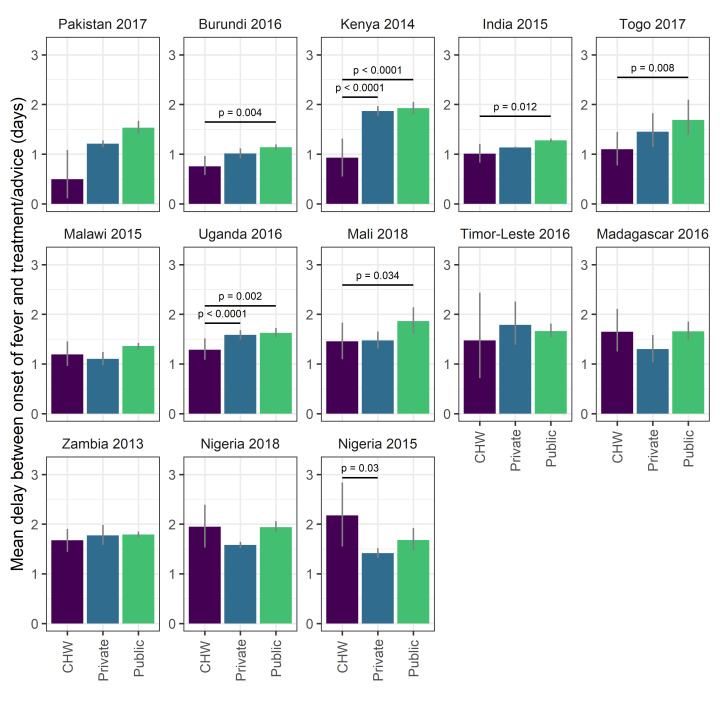
The average delay before treatment (days after fever began that advice or treatment was sought) by treatment provider group for all surveys. Values are survey-weighted mean estimates with 95% weighted-bootstrapped confidence intervals. Horizontal lines and *P*-values indicates significantly different mean times between groups as determined by bootstrapped hypothesis test. Surveys are ordered by descending average treatment delays for community health workers.

Overall 30.2% (95% CI = 26.7%, 33.3%) of visits to CHWs for a fever occurred within 24 hours, similar to the private sector (28.6%, 95% CI = 27.9%, 29.3%) and higher than the public sector (22.7%, 95% CI = 22.0%, 23.4%), although there was considerable variation between countries (Table S3 in the Online Supplementary Document). Mean delays were 1.32 days (95% CI = 1.2, 1.43) for CHWs, 1.28 days (95% CI = 1.26, 1.3) for the private sector and 1.51 days (95% CI = 1.48, 1.54) for the public sector. In two surveys (Kenya, 2014; Uganda, 2016) the mean delay before treatment or advice was sought was significantly shorter for visits to CHWs than to the public or private sector (**Figure 5**). In four further surveys (Burundi, 2016; India, 2015; Mali, 2017; Togo, 2017) the mean delay before treatment or advice was sought was significantly shorter for visits to CHWs than to the public sector and not significantly different than for visits to the private sector. In Nigeria, the mean delay before treatment or advice was sought was significantly longer than the private sector in 2015 and not significantly different from either public or private sectors in 2018, suggesting weak evidence for a positive trend in access to CHWs with respect to time. For the remaining 6 surveys, the mean delay before treatment or advice was sought was not significantly different for visits to CHWs compared to those to the public or private sector.

## DISCUSSION

We identified evidence of CHW activity in 21 countries surveyed. In areas where CHWs were identified as being active, the contribution of CHWs relative to other types of health care providers varied widely but made up at least 10% of all treatment visits and exceed 40% of all treatment visits in some instances (for example 44.8% in Madagascar and 44.3% in Rwanda (where multiple CHWs are stationed in every village [17]) (**Table 1**). These results are broadly in line with previous estimates of the contribution of iCCM in relation to total treatments (27%, 95% CI = 1-74%] [18]. At the country levels contribution was lower but in 21% of surveys (6/29) treatment visits to CHWs contributed to more than 5% of all treatment visits recorded (Table S2 in the **Online Supplementary Document**). The distribution of treatments provided by CHWs was also very different between countries (**Figure 1**) reflecting the diverse epidemiological settings as well as scope of CHW training and implementation in different places. Broadly we see similar patterns of treatment and testing from CHWs as in the public sector (**Figure 1**), however more detailed studies are better equipped to assess quality of care and appropriateness of treatment.

### Equity-focus

Assessing the ability of iCCM programmes to address health inequities in access, utilisation and standards of health care is key to understanding the value of such programmes. All three metrics used here to characterise potentially underserved populations – poverty, rurality and remoteness – indicate that CHWs are in the majority of instances, being successfully targeted to address inequities in health care access. Clear trends are seen with respect to wealth quintile with CHWs playing a more prominent role in diagnosis and treatment to those in poorer quintiles (**Figure 2**). The majority of CHW activity is recorded in rural settings (**Figure 3**). This trend is more clear than trends with respect to remoteness (**Figure 4**) where spatial scale and distribution of urban centres, which vary a lot between countries and likely differ from time to treatment centres, influence the signal. This highlights the need for further work to determine the extent to which successful targeted deployment vs treatment seeking and behavioural factors are driving these associations.

### Treatment delays

There is still the need for much improvement in health care access; for more than two thirds of records the delay between the onset of fever and treatment seeking took longer than the crucial 24 hours after onset that has been set as a target by the WHO and UNICEF [1], although studies have shown CHWs to be associated with reduced delays: in the RAcE project, the percentage of children who received ACT within the same or next day following the onset of fever increased significantly, from 57% at baseline to 74% at endline (*P* < 0.05) [19]. Whilst the majority of treatment seeking from all providers takes longer than the 24hr target, in many instances, treatment from a CHW was obtained significantly faster or not significantly slower (with the exception of older surveys from Nigeria) than from either public or private sources (**Figure 5**). Of note is that this is a country-wide assessment, not confined to those areas where CHWs have been targeted. In some cases, if we assume CHWs are effectively targeted to low-access areas, this may be a conservative estimate in the improvements to treatment delays. Sub-setting the data to consider only those clusters where CHW activity was recorded shows similar patterns and, despite the smaller sample size, in some instances (for example Nigeria 2018) an even greater effect (Figure S4 in **the** **Online Supplementary Document**). Interpretation of these trends is further complicated as treatment seeking behaviour is influenced by severity of symptoms [20]. Severe symptoms and the choice or referral to attend primary, secondary or tertiary health care may confound these results. Evidence from clinical studies may reflect this, showing longer delays to treatment than reported here [9,21-23]. A more robust assessment of the impact of introducing CHWs on treatment delays would include a comparison of delays in a location before and after deployment of CHWs. Unfortunately, we did not recover enough multiple year surveys with CHW information in this analysis to assess temporal changes in access.

### Limitations

The results presented here must be interpreted with a number of important caveats in mind. Of most significance are issues of data completeness. The observed number of CHW records is small for many surveys and the quality and detail of documented treatment providers varied widely. We therefore stress the importance of interpreting results with respect to uncertainty estimates as well as the point estimate. As such there are likely issues in our ability to identify all CHW interactions (sensitivity) as well as the potential for recorded CHW interactions to be erroneously classified (specificity). In some instances, whole programmes are not picked up, for example since 2004 Ethiopia has had a well-established programme of health extension workers (38 000 trained in the first five years [5]), yet due to lack of detail in provider information we cannot discriminate this in the DHS surveys in Ethiopia from 2011 or 2016. Estimates of CHW in Nigeria here are lower than those observed at end line in the RAcE study for Niger state (12.5% compared to 84% percent). This comparison highlights an important point, that in this study it was difficult to distinguish between CHW programmes in general and those providing iCCM specifically. In general, the lack of standardised, multi-country data sets specific to iCCM implementation and the contribution of CHWs make validation of our results difficult and we would advocate for future work to aim to fill this knowledge gap. DHS survey questionnaires are also subject to recall bias which could interact with severity or treatment provider. Furthermore, iCCM programmes and CHW distribution are often sub-nationally implemented or targeted. As such, country-level summaries may not show all of the sub-national heterogeneity in programmes within a country. Whilst DHS surveys are standardised across countries, between country comparison must be interpreted with some caution, especially with respect to differences in the composition of some variables, for example the wealth index.

Our broad classification of providers into five groups simplifies what is, in reality, a more nuanced spectrum of providers. For example, the private classification may range from small local drug shops, proprietary and patent medicine vendors (PPMVs) or corner shops that happen to sell drugs (that usually are accessible and sell their drugs in ways which are affordable for low income households) right up to private clinics and hospitals (that cater for the wealthier section of society). In this case, we lack resolution to see variations in provider, quality of care and the interaction with wealth and country.

Whilst we can assess evidence of coverage, access and equity outcomes, we do not evaluate the quality of care or downstream health outcomes which are beyond the scope of this analysis. Improved access and reduced delays should lead to improved health outcomes [9,21,23] although confounding factors such as quality of care, referral efficiency, treatment seeking behaviour and public/private preferences are also important.

## CONCLUSIONS

There remains much progress to be made in reducing under five mortality, despite improvements in recent years catalysed by the millennium development goals [24]. Access to prompt effective health care must not only be increased but increased in a manner that addresses the deep inequities in access to health care that are currently present. The implementation of iCCM is one such proposed method. Evidence presented here suggests that community health workers are associated with reduced treatment delays and are being successfully targeted to underserved populations. Further efforts to increase the coverage of effective and impactful health care are undoubtedly needed and it is likely that iCCM is and will continue to play a key role in future strategies.

## Additional material

Online Supplementary Document
