# Supplementary material for: Does integrated community case management (iCCM) target health inequities and treatment delays? Evidence from an analysis of Demographic and Health Surveys data from 21 countries in the period 2010 to 2018
Source: J Glob Health. 2021 Mar 1;11:04013. doi: 10.7189/jogh.11.04013 (PMC7979156; doi:10.7189/jogh.11.04013)
Supplement: Online Supplementary Document [file jogh-11-04013-s001.pdf]

Online Supplementary Document: Does integrated community case management (iCCM) target health inequities and treatment delays? Evidence from an analysis of Demographic and Health Surveys data from 21 countries in the period 2010 to 2018.

Winskill, P.[1], Mousa, A.[1], Oresanya, O.[2], Counihan, H.[3], Okell, L[1], Walker, P. G.[1],

1. MRC Centre for Global Infectious Disease Analysis, Imperial College London.
2. Malaria Consortium, Abuja, Nigeria
3. Malaria Consortium, London, UK

Provider classifications

Community Health Worker

Government community health worker, community health worker, community relay, Public: ASHA, (Private) Village Health Worker, government HSA, Community Health worker ("ADBC"), health cabinet/ CHW, village health worker, role model caregiver/community worker, private fieldworker/CHW, public fieldworker/CHW, Lady h.worker, SISCa post, Fieldworker/Community health worker, private community health worker, public fieldworker/VHT, community health worker (VHT), community health worker/VHT, private fieldworker/VHT, private community based agent/fieldworker, public community based agent/fieldworker, Government community health worker, community health worker, community relay, Public: ASHA, (Private) Village Health Worker, government HSA, Community Health worker ("ADBC"), health cabinet/ CHW, village health worker, Lady h.worker, CS community health worker, Community health worker, SISCa post, Private community health worker, Public fieldworker/VHT, public fieldworker/VHT, community based agent/fieldworker

Public

Government health center, Religious Hospital, Government trained health worker, government hospital, Government "Centre Social", Government family planning clinic, Government field worker, government health center, government health post, health worker, other public sector, public health center, national hospital, district hospital, registered health center, regional hospital, registered hospital, mobile nurse, mobile clinic, Public: Govt./Municipal Hospital, Public: Sub-Centre/ANM, Public: CHC/Rural Hospital/Block PHC, Public: PHC/Additional PHC, Public: UHC/UHP/UFWC, Public: Anganwadi/ICDS Centre, NGO or Trust Hospital/Clinic, Public: Govt. Mobile Clinic, Public: Camp, Public: Vaidya/Hakim/Homeopath (AYUSH), Other Public Health, government health center, mission/ hospital clinic, (Public) Government Health Post, government hospital II, health center II, government hospital I, health center I, CHAM / mission hospital, government health post / outreach, CHAM / mission health center, government mobile clinic, Community Health Center ("CSCOM"), Reference Health Centre ("CSREF"), government regional hospital, government national hospital, Other public sector, community health center, reference health center, government health center (RHC), public traditional medical clinic, public mobile clinic, free mobile clinic, BHU(Basic Health Unit), Rural health centre/Mother child health centre, health center, government reference hospital, District hospital, health post, referral hospital, provincial / district hospital, other public facility, government mobile team, public outreach worker, other public, Community health centre, Referral hospital, Health post, National hospital, Mobile clinic, Government health post, Government hospital, public out reach service, PNFP health center, PNFP hospital, outreach/mobile clinic, government health center/post, public mobile hospital/clinic, Government health center, Religious Hospital, Government family planning clinic, government hospital, Government field worker, Government trained health worker, Government "Centre Social", public health center, registered health center, regional hospital, district hospital, national hospital, other public sector, registered hospital, government health post, government health center, mobile nurse, health worker, mobile clinic, Public: CHC/Rural Hospital/Block PHC, Public: Sub-Centre/ANM, Public: UHC/UHP/UFWC, NGO or Trust Hospital/Clinic, Public: PHC/Additional PHC, Public: Govt./Municipal Hospital, Public: Govt. Mobile Clinic, Public: Vaidya/Hakim/Homeopath (AYUSH), Public: Anganwadi/ICDS Centre, Other Public Health, Public: Camp, government dospensary, mission hospital, (Public) Government Health Post, government health post / outreach, government mobile clinic, Community Health Center ("CSCOM"), Reference Health Centre ("CSREF"), government national hospital, government regional hospital, community health center, reference health center, government health center (RHC), other public center, public mobile clinic, public traditional medical clinic, public fieldworker, Rural health

centre/Mother child health centre, BHU(Basic Health Unit), health center, government reference hospital, health post, other public facility, CS health post, provincial / district hospital, Mobile clinic, public outreach worker, other public, Community health centre, National hospital, Referral hospital, Health post, outreach/mobile clinic, government health center/post, mobile hospital/clinic, mission hospital/clinic

Private

private pharmacy, Market, Shop, private hospital/clinic, private doctor, Other private medical, private hospital/ clinic, market, private health worker, shop, private health center, other sector market, other sector shop, other medical private, Private: Hospital, Public: Govt. Dispensary, Private: Doctor/Clinic, Other Private Health, Private: Vaidya/Hakim/Homeopath (AYUSH), Private: Paramedic, Private: Pharmacy/Drugstore, government dispensary, other private medical, (Other) Shop, (Private) CHAL Health Center, (Private) Red Cross Health Center, (Private) CHAL Hospital, (Private) CHAL Health Post, kiosk, private HSA, private mobile clinic, Other private medical sector, Itinerant drug seller, dispensary/maternity clinic, Street vendor, pharmacy, dispensary maternity, street drug vendor, other private, other private sector, chemist/PMV, chemist/PMS, itinerant drug seller, other private medical sector, private pharmacy (chemist/medical store), private polyclinic, dispensary, private doctor/clinic, Kiosk, kiosk / shop, private comm. health worker, private outreach worker, Dispensary, Other private sector, private out reach service, private pharmacy/drug shop, hawker/itinerant drug seller, private mission hosptial/clinic, private mobile hospital/clinic, Market, Shop, private pharmacy, private doctor, private hospital/clinic, private health center, other source itinerant drug seller, other source shop, shop, market, other medical private, Private: Hospital, Private: Doctor/Clinic, Other Private Health, Private: Pharmacy/Drugstore, Private: Paramedic, Public: Govt. Dispensary, Private: Vaidya/Hakim/Homeopath (AYUSH), other private, (Private) CHAL Health Center, (Private) CHAL Health Post, (Private) CHAL Hospital, private CHAM / mission health center, private CHAM / mission hospital, private mobile clinic, other private medical, private HSA, Street vendor, pharmacy, dispensary/maternity clinic, Dispensary/ maternity, street drug vendor, chemist/PMS, private fieldworker, itinerant drug seller, other private medical sector, private pharmacy (chemist/medical store), dispensary, private doctor/clinic, kiosk / shop, other private medical facility, private comm. health worker, private outreach worker, Other private medical sector, private pharmacy/drug shop, other private sector

Traditional

Traditional Practitioner, traditional pract, other source traditional practitioner, traditional pract., Traditional Healer, traditional practitioner, (Other) Traditional Healer, traditional healer, Traditional practitioner, traditinal practitioner, Traditional Practitioner, other source traditional practitioner, traditional pract., Traditional Healer, traditional practitioner, (Other) Traditional Healer, Traditional practitioner, traditional healer, traditional practioner

Other

Friends, relatives (CS other sector), Other, NGO Field Worker, other, maternity clinic, Friend/Relative, relative/ friend, Health Facility Outside Lesotho, friend/family, BLM, Youth drop in centre, Community health clinic ("Salle de soin"), medical care office, Dumba Nengue, NGO - Myanmar Medical Association (MMA), other NGO sector, NGO - Myanmar Red Cross, UHC/MCH center, drug hawker, other pricate sector, community-oriented resource person, Dispenser/compounder, Homeopath, Hakim, DAI/TBA, friend/relative, outreach, church, clinic, Friend / relative, polyclinic, friend / relative, Other, Family Planning Association of Benin (ABPF), Friend/Relative, relative/friend, Household has a lamp/torch, Household has a bed with a mattress, BLM, Household has a paraffin lamp, Household has a Koloboyi, YOUTH DROP CENTER, How often

members of household went to bed hungry, other, How often spent day/night hungry, medical care office, Dumba Nengue, other NGO sector, NGO - PSI/M (SUN), UHC/MCH center, NGO - Myanmar Red Cross, community-oriented resource person, Dispenser/compounder, Homeopath, Community midwife, Hakim, DAI, TBA, friend/relative, outreach, friend / relative, polyclinic

Figures and tables

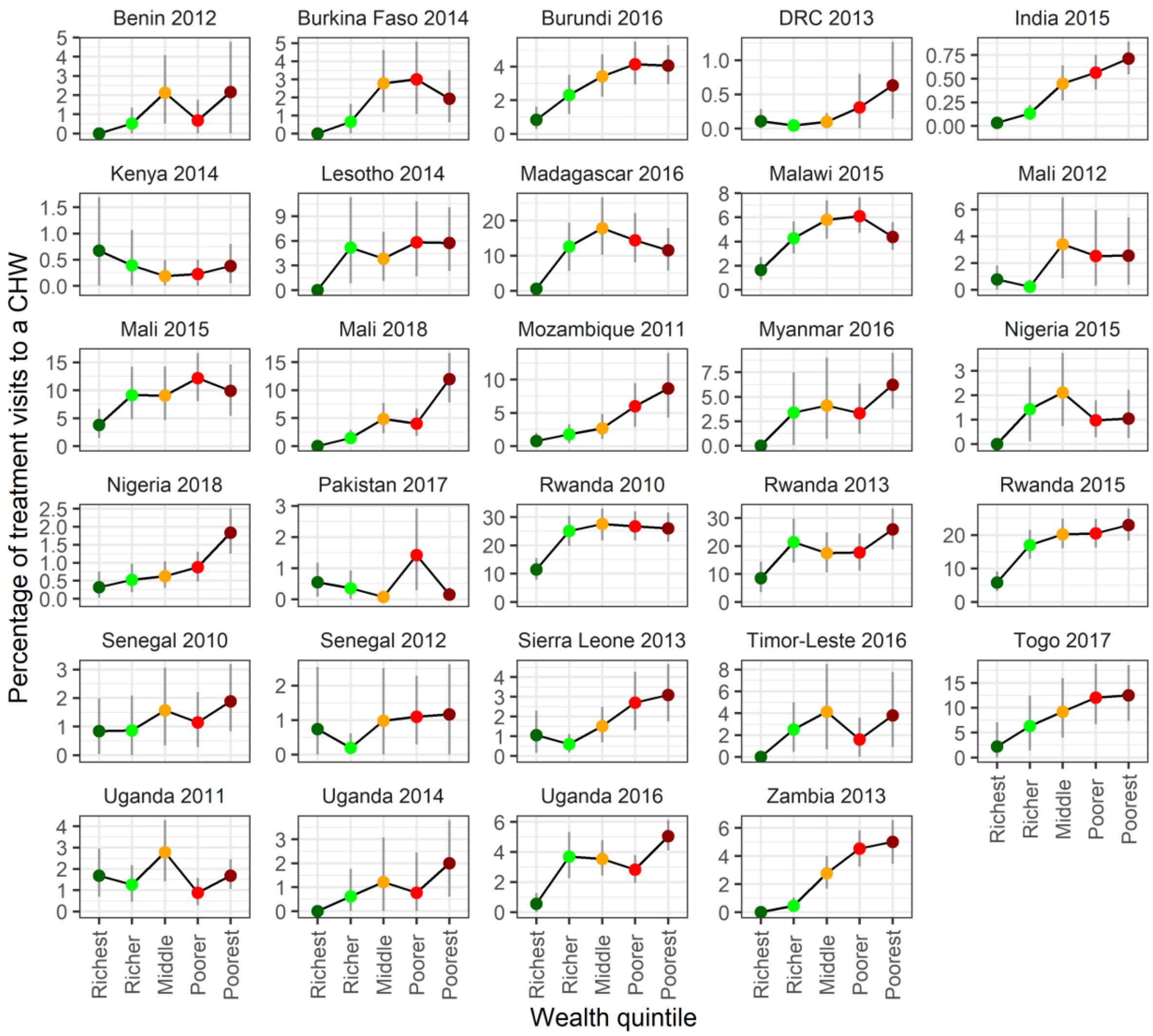

Figure S1. The percentage of treatment visits with a Community Health worker (CHW) as a function of demographic and health survey wealth quintile for all years analysed. There is an increasing trend in the percentage of treatment visits with a CHW as poverty increases. Values are survey-weighted mean estimates with 95% weighted-bootstrapped confidence intervals.

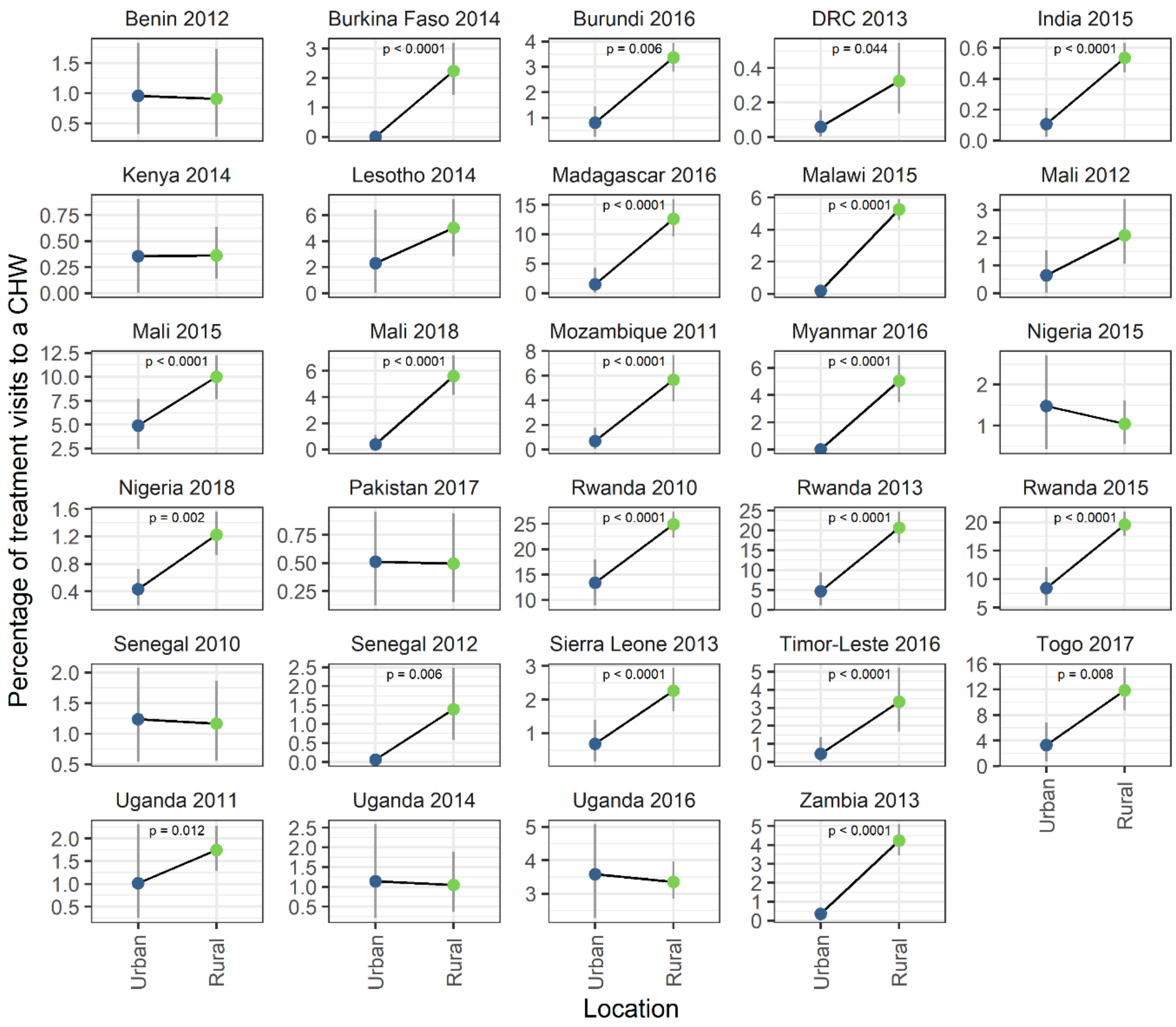

Figure S2. The percentage of treatment visits to a Community Health Worker (CHW) as a function of urban or rural location for all years analysed. Visits to CHWs constitute a higher percentage of treatment visits in rural locations compared to urban locations. Values are survey-weighted mean estimates with 95% weighted-bootstrapped confidence intervals. P-values indicate significantly different mean proportions between groups as determined by bootstrapped hypothesis test.

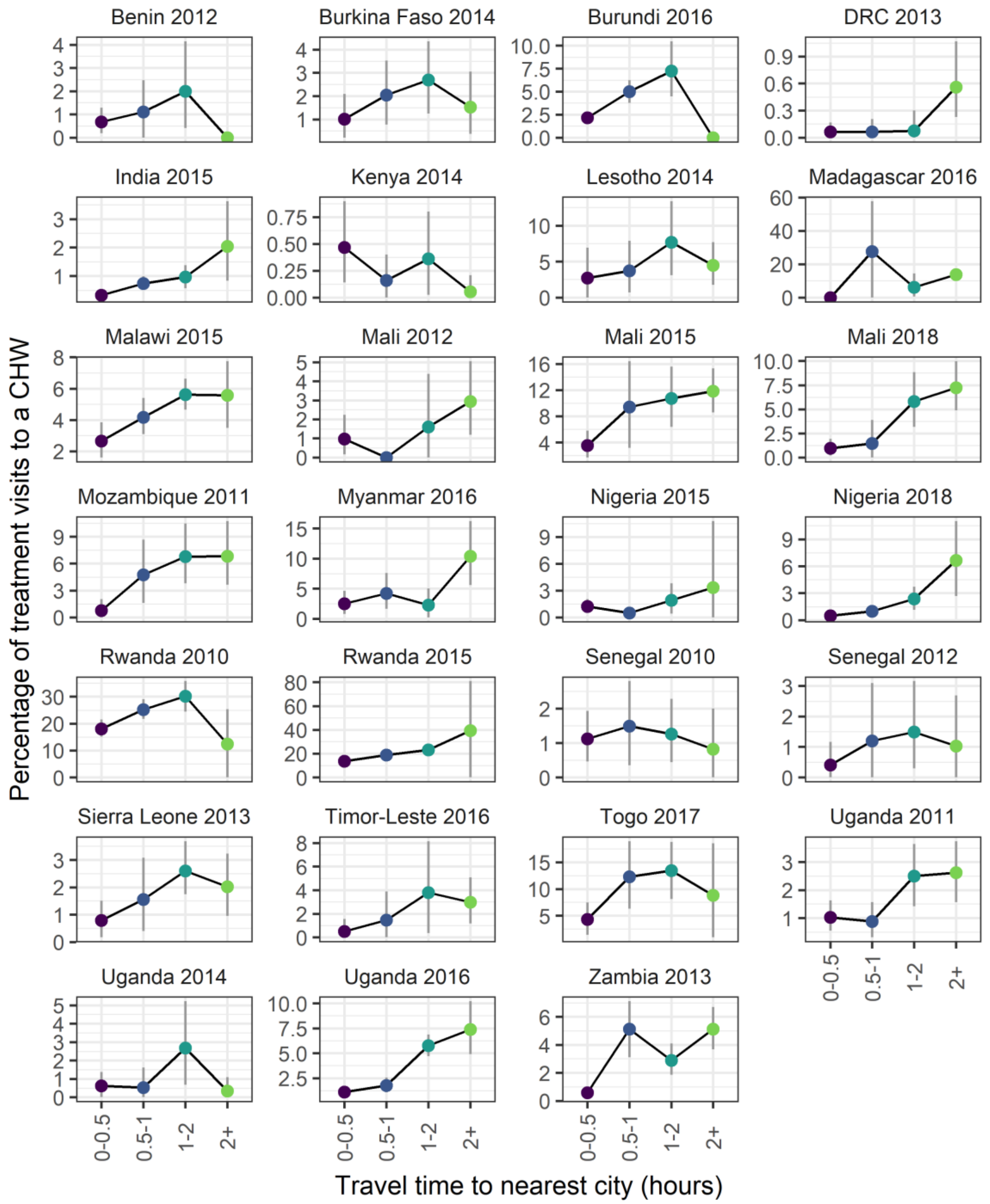

Figure S3. The percentage of treatment visits to a Community Health worker (CHW) as a function of remoteness (travel time to the nearest city) for all years analysed. Values are survey-weighted mean estimates with 95% weighted-bootstrapped confidence intervals. For a number of countries there is an increasing trend in the percentage of treatment visits with a CHW as remoteness increases. P-values indicate significantly different mean proportions between groups as determined by bootstrapped hypothesis test.

Table S1. The percentage of treatment visits assigned to each healthcare provider group for each demographic and health survey. Only clusters where Community Health Worker activity was identified were included. Values are survey-weighted percentages with 95% weighted-bootstrapped confidence intervals.

Country	Year	N	Percentage of visits from each provider (95% CI)				
			CHW	Public	Private	Traditional	Other
**Benin**	2012	49	31.2 (17.6, 45.3)	41.4 (27.5, 56.8)	18.6 (8.7, 29.3)	8.8 (2, 16.9)	0 (-, -)
**Burkina Faso**	2014	183	15.3 (9.9, 20.7)	71.8 (65.1, 78.4)	7.6 (3.8, 11.8)	4.7 (1.8, 8.1)	0.6 (0, 1.8)
**Burundi**	2016	709	18.7 (15.9, 21.6)	74.3 (70.9, 77.8)	6 (4.3, 7.9)	0.6 (0, 1.4)	0.4 (0, 1)
**DRC**	2013	178	10.3 (4.8, 16.9)	53.1 (43.3, 63.3)	27.9 (18.9, 37.5)	7.5 (2.7, 12.7)	1.2 (0, 3.9)
**India**	2015	761	30.6 (26.4, 35)	26.8 (22.8, 31)	39.7 (34.9, 44.1)	0.8 (0, 1.8)	2.2 (1.1, 3.6)
**Kenya**	2014	67	28.5 (14.6, 43.6)	27.7 (14.8, 42.4)	36.6 (22.8, 51.7)	0 (-, -)	7.2 (0, 15.5)
**Lesotho**	2014	70	36.2 (23.7, 51.1)	44.5 (29.1, 58.4)	17.7 (8.6, 28.1)	0 (-, -)	1.7 (0, 4.3)
**Madagascar**	2016	163	44.8 (35.5, 54)	42.5 (33.3, 52)	7.9 (3, 13.8)	1.6 (0.1, 3.7)	3.2 (0, 8.5)
**Malawi**	2015	1325	20.3 (17.9, 22.8)	63.1 (60.2, 66)	12.8 (10.7, 14.9)	0 (0, 0.1)	3.8 (2.6, 5)
**Mali**	2012	55	42.4 (24.5, 58.3)	27.3 (12.6, 43.3)	21.1 (9.7, 33.8)	3.7 (0, 8.9)	5.5 (0, 15)
**Mali**	2015	490	21.1 (17.1, 25.4)	42.5 (37, 47.3)	20.1 (16, 24.3)	15.1 (11.8, 18.8)	1.3 (0.4, 2.4)
**Mali**	2018	257	24.3 (18.9, 30.4)	19.5 (14.4, 25)	46.1 (39, 53.2)	7.3 (3.7, 11.3)	2.8 (0.8, 5.3)
**Mozambique**	2011	121	37.9 (26.7, 49.1)	54.2 (42.6, 66)	1.1 (0, 3.6)	0.7 (0, 2.4)	6.1 (1.5, 12.5)
**Myanmar**	2016	141	36.4 (26, 47.3)	26.4 (18.1, 35.7)	27.5 (17.2, 38.3)	1.4 (0, 3.8)	8.2 (2.5, 15.1)
**Nigeria**	2015	99	22.1 (14.4, 31.3)	23.6 (14.7, 33)	51.6 (41.5, 61.7)	0.8 (0, 2.1)	1.9 (0, 7.1)
**Nigeria**	2018	713	12.5 (9.4, 15.6)	36.1 (32.2, 40.1)	49.8 (45.7, 54)	1 (0.2, 2)	0.6 (0.2, 1)
**Pakistan**	2017	168	11.1 (5.7, 18.1)	15.4 (9.3, 22.7)	67.5 (58.3, 76.4)	0 (-, -)	5.9 (1.7, 11)
**Rwanda**	2010	849	41.7 (38.3, 45.3)	36.9 (33.6, 40.4)	7 (5.2, 9)	2.7 (1.6, 4.1)	11.7 (9.5, 14.1)
**Rwanda**	2013	307	36 (30.4, 42)	45.4 (39, 50.9)	7.4 (4.7, 10.7)	2.9 (1.2, 4.9)	8.4 (5.4, 11.6)
**Rwanda**	2015	777	40.3 (36.5, 44)	45.2 (41.6, 49)	10.2 (7.8, 12.5)	1 (0.3, 1.7)	3.4 (2.2, 4.7)
**Senegal**	2010	258	12.7 (8.1, 18.4)	68.5 (61.3, 75.2)	16 (10.1, 22.7)	1.4 (0.2, 3.3)	1.3 (0.3, 2.6)
**Senegal**	2012	109	13.9 (6.8, 22.9)	65.9 (54.5, 77)	15.8 (6.9, 25.7)	4 (0, 9.2)	0.5 (0, 1.6)
**Sierra Leone**	2013	385	17.1 (12.5, 21.6)	73 (67.8, 78.1)	5.2 (3, 7.9)	2.5 (1, 4.1)	2.2 (0.7, 3.9)
**Timor-Leste**	2016	48	43 (24.9, 61.7)	51.6 (34, 68.8)	3.6 (0, 11.5)	1.8 (0, 6.4)	0 (-, -)
**Togo**	2017	175	27.2 (20.4, 34.4)	12.8 (7.3, 18.7)	53.1 (45.6, 60.3)	3.8 (1.4, 6.5)	3.1 (0.8, 5.9)
**Uganda**	2011	600	13.4 (10.3, 17)	32 (27.5, 36.4)	51.6 (46.4, 56.3)	0.1 (0, 0.2)	2.9 (1, 5.3)
**Uganda**	2014	90	16.2 (7.7, 26.1)	32.2 (21, 43.7)	50.3 (37.7, 62.3)	1.3 (0, 4)	0 (-, -)
**Uganda**	2016	1189	20 (17.3, 22.9)	40.1 (36.7, 43.7)	39.1 (35.6, 42.8)	0.5 (0.1, 1.1)	0.3 (0, 0.6)
**Zambia**	2013	379	28.2 (23.5, 33)	60.6 (55.5, 65.8)	7.1 (4.1, 10.4)	2.5 (0.8, 4.5)	1.6 (0.3, 3.3)

Table S2. The country-level percentage of treatment visits assigned to each healthcare provider group for each demographic and health survey. Values are survey-weighted percentages with 95% weighted-bootstrapped confidence intervals. In a number of countries Community Health Workers constitute a very low proportion of total treatments provided.

Country	Year	N	CHW	Public	Private	Traditional	Other
Benin	2012	1363	0.9 (0.5, 1.5)	52.2 (49.4, 55.4)	35.7 (32.7, 38.3)	6.5 (5.2, 7.9)	4.5 (3.2, 6)
Burkina Faso	2014	1693	1.8 (1.1, 2.5)	87.9 (86.2, 89.7)	6.4 (5, 7.7)	2.5 (1.7, 3.3)	1.4 (0.7, 2.2)
Burundi	2016	4241	3.2 (2.7, 3.7)	85.4 (84.3, 86.6)	10.3 (9.4, 11.3)	0.5 (0.3, 0.8)	0.6 (0.4, 0.9)
DRC	2013	5784	0.2 (0.1, 0.4)	54.4 (52.5, 56.1)	38.6 (36.8, 40.4)	4.3 (3.6, 5.1)	2.5 (1.9, 3.1)
India	2015	45148	0.4 (0.3, 0.5)	23 (22.5, 23.6)	72.3 (71.7, 72.9)	0.5 (0.4, 0.6)	3.8 (3.6, 4)
Kenya	2014	6660	0.4 (0.2, 0.6)	42.5 (40.9, 44.1)	56 (54.3, 57.5)	0.4 (0.3, 0.7)	0.8 (0.5, 1.1)
Lesotho	2014	681	4.3 (2.6, 6.2)	58.2 (53.9, 63)	33.7 (29.5, 38.1)	1.2 (0.4, 2.1)	2.7 (1.4, 4.2)
Madagascar	2016	639	11.5 (8.7, 14.5)	59.7 (54.6, 64.1)	21.5 (17.4, 25.6)	1.6 (0.7, 2.7)	5.8 (3.6, 8.3)
Malawi	2015	5630	4.6 (4, 5.2)	74.4 (72.9, 75.8)	14.9 (13.6, 16.2)	0.2 (0.1, 0.4)	5.8 (5, 6.5)
Mali	2012	1020	1.7 (0.9, 2.6)	45.2 (41.1, 48.9)	33 (29.7, 36.5)	14.4 (11.7, 17.2)	5.8 (3.9, 7.7)
Mali	2015	1198	8.8 (6.9, 10.8)	50.6 (47.3, 54)	25.4 (22.7, 28.4)	13.8 (11.6, 16)	1.3 (0.7, 2)
Mali	2018	1613	4.4 (3.3, 5.6)	33 (30.5, 35.6)	49.7 (46.9, 52.5)	8.9 (7.3, 10.4)	4 (3, 5)
Mozambique	2011	1523	4 (2.7, 5.2)	89.4 (87.4, 91.4)	1.5 (0.9, 2.2)	2.6 (1.6, 3.8)	2.5 (1.6, 3.5)
Myanmar	2016	1176	3.8 (2.5, 5.4)	53.4 (49.7, 57.2)	38.1 (34.4, 42)	1.3 (0.5, 2.3)	3.4 (2.1, 4.7)
Nigeria	2015	1752	1.2 (0.7, 1.7)	29.3 (26.9, 31.7)	66.6 (64.2, 69.1)	1.8 (1.2, 2.5)	1.2 (0.7, 1.7)
Nigeria	2018	8120	1 (0.8, 1.2)	35.1 (33.9, 36.3)	60.8 (59.5, 62)	1.8 (1.5, 2.1)	1.3 (1, 1.6)
Pakistan	2017	5214	0.5 (0.2, 0.8)	17.1 (15.6, 18.6)	72.8 (71, 74.6)	0 (-, -)	9.6 (8.4, 10.8)
Rwanda	2010	1604	23.1 (20.8, 25.2)	50.6 (47.8, 53.2)	11.7 (10.1, 13.4)	3.8 (2.8, 4.8)	10.9 (9.2, 12.5)
Rwanda	2013	622	18.3 (15.4, 21.2)	53.4 (49.4, 57.4)	13 (10.1, 15.8)	3.3 (1.9, 4.9)	12 (9.6, 14.9)
Rwanda	2015	1801	17.5 (15.6, 19.3)	58.8 (56.3, 61)	15.4 (13.7, 17.3)	2.6 (1.8, 3.4)	5.8 (4.7, 7)
Senegal	2010	2606	1.2 (0.8, 1.7)	69.6 (66.9, 72.2)	20.8 (18.4, 23.1)	4 (3.1, 4.9)	4.5 (3.3, 5.9)
Senegal	2012	1206	0.8 (0.4, 1.3)	71.8 (68.1, 75.6)	23 (19.3, 26.5)	3.2 (2.1, 4.6)	1.2 (0.6, 1.9)
Sierra Leone	2013	3337	1.8 (1.3, 2.4)	83.7 (82.1, 85.2)	11.3 (10, 12.6)	1 (0.7, 1.4)	2.2 (1.6, 2.8)
Timor-Leste	2016	1055	2.1 (1.1, 3.2)	87.8 (85.1, 90.4)	8.9 (6.6, 11.2)	0.7 (0.2, 1.3)	0.5 (0, 1.2)
Togo	2017	461	9 (6.6, 11.5)	22.4 (17.8, 27.2)	63.9 (58.5, 68.9)	2.2 (1, 3.6)	2.5 (1.3, 4.1)
Uganda	2011	4530	1.6 (1.2, 2.1)	37.1 (35.5, 38.8)	59 (57.3, 60.5)	1 (0.5, 1.5)	1.3 (0.9, 1.7)
Uganda	2014	1126	1.1 (0.4, 1.8)	45.5 (42.2, 48.7)	52.9 (49.6, 56.5)	0.2 (0, 0.4)	0.3 (0.1, 0.7)
Uganda	2016	5783	3.4 (2.9, 3.9)	40.7 (39.2, 42.1)	54.6 (53.1, 56.2)	0.7 (0.5, 1)	0.6 (0.4, 0.8)
Zambia	2013	3896	2.9 (2.3, 3.5)	87 (85.8, 88.2)	8.6 (7.6, 9.7)	0.8 (0.5, 1.1)	0.7 (0.4, 1)
Total	NA	121482	2.1 (2, 2.2)	42.6 (42.3, 43)	50.6 (50.2, 50.9)	1.5 (1.4, 1.6)	3.2 (3.1, 3.3)

Table S3. Treatment seeking delays. The percentage (95% bootstrapped confidence intervals) of treatment seeking visits for fever to each type of provider (Community Health Worker (CHW), public and private providers) that occur within a given timeframes.

		0-24 hours			24-48 hours		
**Country**	**Year**	**CHW**	**Public**	**Private**	**CHW**	**Public**	**Private**
Burundi	2016	51.5% (43.4%, 59.7%)	33.1% (31.1%, 34.8%)	35.5% (29.8%, 40.8%)	33.5% (25.6%, 41.5%)	40.3% (38.5%, 42.3%)	35.8% (29.9%, 41.5%)
India	2015	31.0% (20.1%, 41.4%)	25.2% (23.9%, 26.7%)	29.6% (28.8%, 30.5%)	45.0% (34.2%, 56.2%)	40.1% (38.4%, 41.8%)	41.3% (40.3%, 42.2%)
Kenya	2014	17.0% (0.0%, 53.9%)	10.8% (8.2%, 13.7%)	11.1% (9.1%, 13.2%)	73.0% (33.0%, 100.0%)	34.3% (30.4%, 38.3%)	34.4% (31.7%, 37.6%)
Madagascar	2016	19.7% (8.7%, 31.0%)	19.4% (13.6%, 25.7%)	30.0% (21.2%, 40.9%)	28.3% (17.3%, 41.6%)	30.4% (24.4%, 36.5%)	36.3% (25.9%, 46.4%)
Malawi	2015	29.8% (22.4%, 37.4%)	23.4% (21.6%, 25.1%)	35.9% (31.0%, 41.6%)	40.7% (32.6%, 48.8%)	43.8% (41.6%, 46.1%)	34.8% (29.5%, 40.3%)
Mali	2018	21.8% (9.1%, 34.7%)	24.6% (19.4%, 29.9%)	28.8% (24.2%, 33.4%)	33.7% (18.7%, 48.9%)	21.8% (17.3%, 26.4%)	26.0% (21.6%, 30.6%)
Nigeria	2015	16.3% (3.5%, 34.6%)	34.1% (29.2%, 38.6%)	36.3% (33.4%, 39.4%)	10.5% (0.0%, 24.9%)	18.3% (14.4%, 22.0%)	20.3% (17.5%, 22.9%)
Nigeria	2018	14.5% (7.2%, 23.5%)	14.5% (12.8%, 16.3%)	23.1% (21.4%, 24.8%)	34.5% (24.1%, 46.4%)	32.2% (29.9%, 34.5%)	31.4% (29.6%, 33.1%)
Pakistan	2017	69.4% (35.3%, 91.4%)	19.2% (15.1%, 23.8%)	30.6% (28.0%, 33.1%)	15.9% (0.0%, 41.2%)	35.8% (30.7%, 41.4%)	38.6% (35.8%, 41.3%)
Timor-Leste	2016	34.7% (0.0%, 64.3%)	19.1% (15.4%, 23.1%)	14.4% (4.9%, 27.4%)	21.3% (0.0%, 50.8%)	30.4% (25.6%, 35.3%)	29.1% (13.8%, 44.3%)
Togo	2017	36.9% (23.7%, 51.9%)	13.8% (6.3%, 22.0%)	31.7% (25.9%, 37.8%)	38.2% (24.5%, 52.2%)	49.6% (37.5%, 61.6%)	39.2% (32.6%, 45.4%)
Uganda	2016	33.5% (25.9%, 41.2%)	26.8% (24.7%, 29.0%)	31.4% (29.2%, 33.7%)	29.3% (21.4%, 37.2%)	29.9% (27.8%, 32.1%)	26.4% (24.4%, 28.4%)
Zambia	2013	12.3% (6.1%, 19.7%)	8.3% (7.1%, 9.6%)	17.5% (12.4%, 22.6%)	34.7% (24.7%, 45.6%)	40.0% (37.8%, 42.3%)	32.1% (25.9%, 39.0%)
Total	-	30.2% (26.7%, 33.3%)	22.7% (22.0%, 23.4%)	28.6% (27.9%, 29.3%)	34.9% (31.5%, 38.3%)	37.6% (36.8%, 38.4%)	37.7% (37.0%, 38.3%)

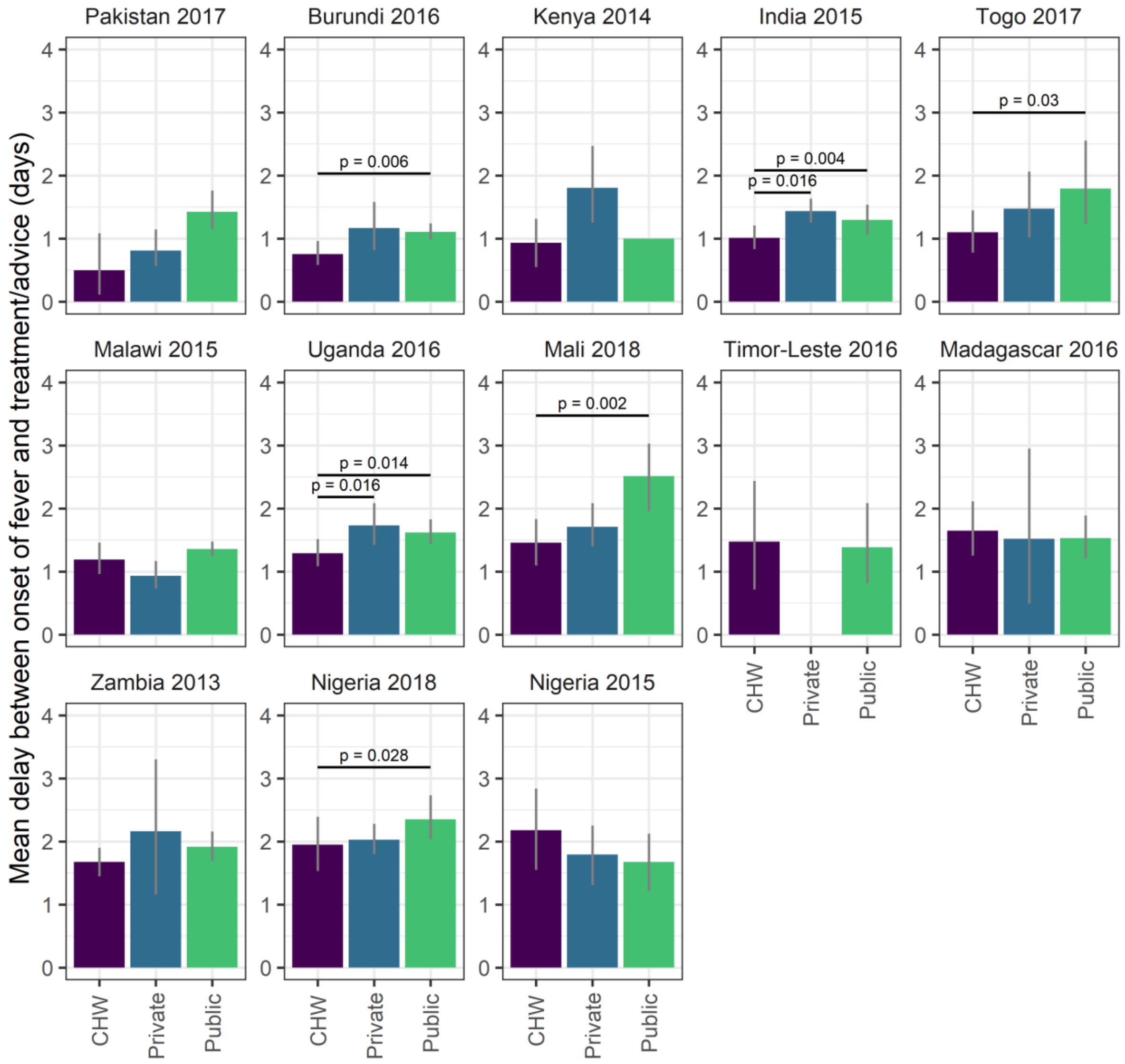

Figure S4. The average delay before treatment (days after fever began that advice or treatment was sought) by treatment provider group with data subset to consider only those clusters where Community Health Worker (CHW) activity was recorded. Values are survey-weighted mean estimates with 95% weighted-bootstrapped confidence intervals. Horizontal lines and p-values indicates significantly different mean times between groups as determined by bootstrapped hypothesis test. Surveys are ordered by descending average treatment delays for CHWs.
